# Synovial mesenchymal stem cell-derived exosomal miR-485-3p relieves cartilage damage in osteoarthritis by targeting the NRP1-mediated PI3K/Akt pathway: Exosomal miR-485-3p relieves cartilage damage.

**DOI:** 10.1016/j.heliyon.2024.e24042

**Published:** 2024-01-03

**Authors:** Mingjun Qiu, Yanhua Xie, Guanghua Tan, Xiaoxu Wang, Peiguan Huang, Liang Hong

**Affiliations:** a Department of joint surgery, The Second Affiliated Hospital of University of South China, China; b Department of orthopedic, The Second Affiliated Hospital of University of South China, China

**Keywords:** Osteoarthritis, Cartilage damage, Exos, miR-485-3p, NRP1

## Abstract

Osteoarthritis (OA) is an age-related musculoskeletal disease that results in pain and functional disability. Stem cell therapy has been considered as a promising treatment for OA. In this study, the therapeutic action and potential mechanism of synovial mesenchymal stem cells (SMSCs)-derived exosomes (Exos) in OA cartilage damage were investigated. Cartilage cells were stimulated with IL-1β to establish an *in vitro* model of OA cartilage damage. Cartilage cell functions were detected by CCK-8, scratch assay, and flow cytometry, respectively. Inflammatory cytokine levels were assessed by ELISA. Target molecule levels were measured by qRT‒PCR and Western blotting. Exos-induced differential expression of miRNAs in cartilage cells were analyzed by microarray analysis. The interaction between miR-485-3p and neuropilin-1 (NRP1) was validated by dual luciferase reporter and RIP assays. We found that treatment with Exos promoted proliferation, migration, and ECM secretion, but restrained apoptosis and inflammation of IL-1β-exposed cartilage cells via up-regulation of miR-485-3p. Additionally, miR-485-3p directly targeted NRP1 to repress NRP1 expression, which subsequently caused inactivation of the PI3K/Akt pathway. The protective effect of Exos on cartilage damage was counteracted by NRP1 overexpression-mediated activation of the PI3K/Akt pathway. In conclusion, Exos delivered miR-485-3p to attenuate IL-1β-induced cartilage degradation by targeting NRP1 and succedent inactivation of the PI3K/Akt pathway. Our findings shed light on the novel protective mechanism of Exos in OA, which suggest that the restoration of miR-485-3p by Exos might be a novel approach for OA treatment.

## 1 Introduction

Osteoarthritis (OA) is a prevalent musculoskeletal inflammatory disease characterized by cartilage damage and joint pain [1]. There is an increasing incidence of OA worldwide, which represents a threat to patients' quality of life and an increased burden on health systems [2]. There are many factors associated with OA, including age, obesity, trauma, and chronic low-grade inflammation [3]. Currently, nonsteroidal anti-inflammatory drugs (the first-line therapy for OA), oral vitamin C and vitamin D, biologics such as platelet-rich plasma, bone marrow aspirate concentrate, and mesenchymal stem cells can only relieve short-term clinical symptoms and cannot slow the progression of OA [4]. Therefore, there is an urgent medical need to develop more effective therapeutic interventions for OA.

Exosomes (Exos) are nanoscale vesicles derived from different types of cells, with a diameter range from 40 to 100 nm. Exos carry multiple biological molecules, such as proteins, lipids, and RNAs, and transport these molecules between cells, thus mediating intercellular communication [5]. Exos may be used for therapeutic purposes in a similar manner to their parental cells [6]. As compared with whole cell-based therapies, mesenchymal stem cells (MSCs)-derived Exos may offer specific advantages for patient safety such as immunoreactivity and no potential of tumor formation [7]. Furthermore, isolation of Exos from MSCs is potentially sustainable and reproducible. Recent studies have suggested that Exos derived from MSCs exert crucial roles in OA [8]. Emerging evidence has indicated that MSC-derived Exos can affect cartilage regeneration by inhibiting chondrocyte hypertrophy and promoting angiogenesis [9]. However, the regulatory mechanism of MSC-derived Exos on cartilage damage in OA remains largely unknown.

It has been recognized that abnormal gene expression in chondrocytes is involved in the pathogenesis of OA [10]. Recently, microRNAs (miRNAs) have been identified to be frequently dysregulated in OA [11]. For instance, miRNA-140 was downregulated in osteoarthritic articular cartilage, which affected the development of OA [12]. Another study found that miR-92a-3p delivered by MSC-derived Exos facilitated cartilage repair, thereby delaying OA progression [13]. miR-485-3p is expressed at low levels in osteoporotic postmenopausal women and has a close correlation with vertebral fractures [14]. More importantly, miR-485-3p could promote proliferation and suppress apoptosis of osteoarthritic chondrocytes [15]. To date, whether miR-485-3p can be transferred by MSC-derived Exos and affect OA progression has not been clarified.

MiRNAs exert their biological functions by regulating gene expression through binding to the 3′-untranslated regions (3′-UTRs) of mRNAs. Neuropilin-1 (NRP1), a transmembrane protein, is widely expressed in multiple tissues and regulates the development of diverse diseases [16]. NRP1 is an axonal guidance molecule that has been recently implicated in regulating bone metabolism [17]. Notably, NRP1 was found to be highly expressed in the cartilage of OA patients [18], suggesting the involvement of NRP1 in cartilage damage during OA. Furthermore, a recent study documented that NRP1 knockdown inhibited MMP13 transcription accompanied by inactivation of PI3K/AKT pathway, which delayed OA chondrocyte proliferation [19]. Interestingly, NRP1 was predicted to be a target gene of miR-485-3p. Therefore, we speculated that exosomal miR-485-3p might affect OA progression by targeting NRP1.

This study explored the potential role of MSC-derived exosomal miR-485-3 in OA. Our results demonstrated that miR-485-3p released by MSC-derived Exos ameliorated cartilage injury during OA by targeting NRP1 to inactivate the PI3K/Akt pathway. Our findings may provide novel evidence that MSC-derived Exos exhibit better therapeutic effects on OA.

## 2 Methods

### 2.1 Identification of SMSCs

Surface antigen expression was detected by flow cytometry to identify SMSCs. The SMSCs were incubated with anti-CD44 (ab243894, Abcam, UK), anti-CD73 (ab288154, Abcam), anti-CD34 (ab81289, Abcam), or anti-CD45 (ab10558, Abcam) antibodies for 30 min at 4 °C in the dark. Then, the labeled SMSCs were detected on a flow cytometer (Agilent, USA).

For determination of the osteogenic differentiation potential, the SMSCs were cultured in osteogenic medium (DMEM/F-12 containing 10 mM b-glycerophosphate, 50 μg/mL ascorbate, and 1 nM dexamethasone) for 21 d, followed by staining with Alizarin Red S Solution (Solarbio, Beijing, China) according to a previous study [20]. For chondrogenic differentiation, SMSCs were induced in SMSC Cell Chondrogenic Differentiation Basal Medium (Cyagen, Suzhou, China) for 14 d and then stained with the Alcian Blue Stain Kit (Solarbio). For evaluation of adipogenic differentiation potential, SMSCs were cultured in adipogenic medium (DMEM/F-12 containing 100 nM dexamethasone, 10 μg/mL insulin, 50 mM indomethacin, and 500 μM isobutylmethylxanthine) for 21 d as previously reported [20]. Lipid droplet formation was observed by the Oil Red O Stain Kit (Solarbio).

### 2.2 Isolation of Exos from SMSCs and identification

Exos were isolated from the conditioned medium of SMSCs as previously described [21] with some alterations. In brief, after culture in serum-free DMEM/F-12 for 48 h, the cell supernatants were collected and centrifuged at 2000×*g* for 10 min, followed by filtration through 0.22 μm filters. After centrifugation at 4000*g* twice, the isolated Exos were ultracentrifuged at 100 000×*g* for 1 h. The final pellets were resuspended in 100 μl of sterile PBS and stored at −80 °C.

The morphology of the extracted Exos was observed using transmission electron microscopy (TEM, Hitachi, Japan). Subsequently, the exosome size was analyzed using dynamic light scattering analysis. In addition, biomarkers of Exos (including CD81 and TSG101) were detected by Western blotting.

### 2.3 Exosome uptake

For analysis of the internalization of Exos, the exosome suspension (100 μL) was mixed with 1 mL of PKH26 dye (Sigma-Aldrich). Then, the cartilage cells were seeded into 6-well plates at a density of 2x10^5^ cells per well, and labeled Exos were added. After incubation for 6 h, the cartilage cells were stained with DAPI. Under fluorescence microscopy, the internalization of Exos by SMSCs was observed.

### 2.4 Cell culture and treatment

Mouse synovial-derived mesenchymal stromal cells (SMSCs, CP-M236), mouse cartilage cells (CP-M087), and mouse primary chondrocytes (CP-M092) were purchased from Procell (Wuhan, China) and cultured in α-MEM (Thermo Fisher, USA) or DMEM/F-12 containing 10 % fetal bovine serum (FBS, Thermo Fisher). Cartilage cells or mouse primary chondrocytes were exposed to IL-1β (10 ng/mL, Sigma‒Aldrich, USA) for 48 h to induce osteoarthritic cartilage injury *in vitro* as previously described [22]. Cartilage cells or mouse primary chondrocytes (1.0 × 10^6^ cells/well) were pretreated with Exos for 2 h and then stimulated with IL-1β. For inactivation of the PI3K/Akt pathway, cartilage cells were treated with 25 μM PI3K-IN-1 (an inhibitor of PI3K, MCE, USA).

### 2.5 Cell transfection

The miR-485-3p inhibitor, inhibitor negative control (NC), miR-485-3p mimic, mimic NC, overexpression plasmid for NRP1 (oe-NRP1), and oe-NC were purchased from GenePharma (Shanghai, China). SMSCs or cartilage cells were seeded into 6-well plates (1.5 x10^6^ cells per well) and transfected with the above segments using Lipofectamine 3000 (Thermo Fisher).

### 2.6 Quantitative real-time polymerase chain reaction (qRT‒PCR)

Total RNA was extracted from cartilage cells or chondrocytes using TRIzol reagent (Thermo Fisher). cDNA was obtained using the ReverTra Ace® qPCR RT Kit (Toyobo, Japan). The relative levels of target genes were measured by qRT‒PCR using SYBR® Green Real-time PCR Master Mix (Toyobo). GAPDH or U6 served as the internal control. The relative gene expression level was analyzed using the 2^−ΔΔCt^ method. The primer sequences are shown in Table 1.

**Table 1:** Oligonucleotide primer sets for qPCR.

Name	Sequence (5'¬3′)	Length
miR-485-3p F	AGGCTGGCCGTGATGAAT	18
miR-485-3p R	GAACATGTCTGCGTATCTC	19
NRP1 F	GACAAATGTGGCGGGACCATA	21
NRP1 R	TGGATTAGCCATTCACACTTCTC	23
ARNT2 F	ACCCGAAGAAGATGCTGATGTC	22
ARNT2 R	TGCCTGCTGTTGCTGAAGTTG	21
CPNE8 F	CGCCGTACACCCCTCCTA	18
CPNE8 R	GTGTGAGGGACATCAGCATCTG	22
ZBTB43 F	AGCATCATGGCTCATAGGCGCT	22
ZBTB43 R	TCAGTGACCTGGTGCTCATCGT	22
HNF4G F	GCAAGCCTTGCAGCTGACTGCGA	23
HNF4G R	CATTCCACCAAGACTAAGAGCTGT	24
GAPDH F	CAATGACCCCTTCATTGACC	20
GAPDH R	TTGATTTTGGAGGGATCTCG	20
U6 F	CAAGGATGACACGCAAA	17
U6 R	TCAACTGGTGTCGTGG	16

### 2.7 Western blotting

Total protein was isolated using RIPA buffer (Beyotime, Haimen, China). The protein concentration was determined using a BCA kit (Beyotime). The protein samples were separated through SDS‒PAGE. Then, the separated proteins were transferred to polyvinylidene difluoride membranes. After blocking in 5 % nonfat dried milk, the membranes were incubated with primary antibodies against CD81 (ab109201, 1:1000, Abcam), TSG101 (bsm-52746R, 1:500, Bioss, Beijing, China), Aggrecan (sc-166951, 1:500, Santa Cruz, USA), Collagen II (bs-11929R, 1:1000, Bioss), MMP13 (bs-10581R, 1:500, Bioss), NRP1 (ab184783, 1:1000, Abcam), PI3K (ab32089, 1:1000, Abcam), p-PI3K (ab182651, 1:500, Abcam), Akt (bs-6951R, 1:500, Bioss), *p*-Akt (bsm-52129R, 1:500, Bioss), or GAPDH (bs-2188R, 1:2000, Bioss) at 4 °C overnight. After incubation with secondary antibodies at 25 °C for 1 h, the protein bands were detected by ECL Western Blotting Substrate (Solarbio). The blots were cut prior to hybridisation with antibodies during blotting.

#### 2.7.1 Cell Counting Kit-8 (CCK-8)

The proliferation of cartilage cells was measured using the CCK-8 Kit (MCE). Briefly, cartilage cells were seeded into 96-well plates (2000 cells per well). Then, 10 μL of CCK-8 solution was added to each well and incubated at 37 °C for 1 h. The absorbance was recorded at 450 nm on a microplate reader (Tecan, Switzerland).

### 2.8 Apoptosis detection

Cartilage cells were resuspended in binding buffer. After staining with Annexin V-FITC for 10 min and incubation with propidium iodide (PI) at 25 °C in the dark, the percentage of apoptosis was evaluated on a flow cytometer.

### 2.9 Wound healing assay

Cartilage cells were seeded on 6-well plates at a density of 1.0 × 10^6^ cells/well. After confluence and serum starvation overnight, a straight scratch was made using a 200 μL pipette tip. After the detached cells were washed away, the cells were subsequently incubated for another 24 h at 37 °C. Wound healing photographs were taken at 0 and 24 h after scratching under an inverted microscope. The migratory ratio was quantitively analyzed using ImageJ software.

## 3 ELISA

The levels of IL-6 and TNF-α in the supernatant of cartilage cells were assessed using the Mouse IL-6 ELISA Kit (Cat. No.: ab100713, Abcam) and Mouse TNF-α ELISA Kit (Cat. No.: ab208348, Abcam) according to the manufacturer's protocols.

### 3.1 Prediction analysis

Online databases starBase (http://starbase.sysu.edu.cn/index.php), miRDB (http://www.mirdb.org/), miRWalk (http://129.206.7.150/), DIANA-microT (http://diana.pcbi.upenn.edu/DIANA-microT), and TargetScan (http://genes.mit.edu/targetscan) were adopted to predict the potential targets of miR-miR-485-3p before the start of the experiment in Jan. 2022.

### 3.2 Microarray analysis

The differential expression of miRNAs in IL-1β-treated cartilage cells with or without treatment with SMSC-derived Exos was examined by miRNA microarray assays. After total RNA isolation using TRIzol, the miRNAs were labeled with the miRCURY™ Hy3™/Hy5™ Power labeling kit (Exiqon, Denmark). Subsequently, hybridization on the miRCURYTM LNA array (v.19.0) (Exiqon) was carried out. After washing, the hybridized sequences were scanned with the Affymetrix GeneChip 7G Microarray Scanne. The experimental data were analyzed by Affymetrix Expression Console Software. The differentially expressed miRNAs with a fold change ≥2.0 were presented using hierarchical clustering.

### 3.3 Dual luciferase reporter assay

The wild-type (WT) or mutant (MUT) sequences of the NRP1 3′UTR containing the putative miR-485-3p binding sites were inserted into the psiCHECK™-2 vector (Promega, USA). Then, the cartilage cells were co-transfected with NRP1-WT or NRP1-MUT plasmid together with miR-485-3p mimic or mimic NC using Lipofectamine 2000 (Thermo Fisher). After transfection for 48 h, the luciferase activity was determined using the Dual-Lucy Assay Kit (Solarbio).

### 3.4 RNA immunoprecipitation (RIP) assay

The direct binding between miR-485-3p and NRP1 was verified by RIP assays using the EZ-Magna RNA immunoprecipitation kit (Millipore) according to a previous study [23]. Briefly, the cartilage cell lysates were incubated with RNA magnetic beads that were preconjugated with anti-Ago 2 (Cell Signaling Technology, USA) or anti-IgG. Finally, the levels of miR-485-3p and NRP1 in the precipitated RNAs were measured by qRT‒PCR.

### 3.5 Statistical analysis

Data are shown as the mean ± standard deviation (SD). Student's *t*-test or one-way analysis of variance (ANOVA) followed by Tukey's test was performed using GraphPad Prism 7 software. A *P* value less than 0.05 was considered statistically significant.

## 4 Results

### 4.1 Identification of SMSCs and their Exos

**Fig. 1:**
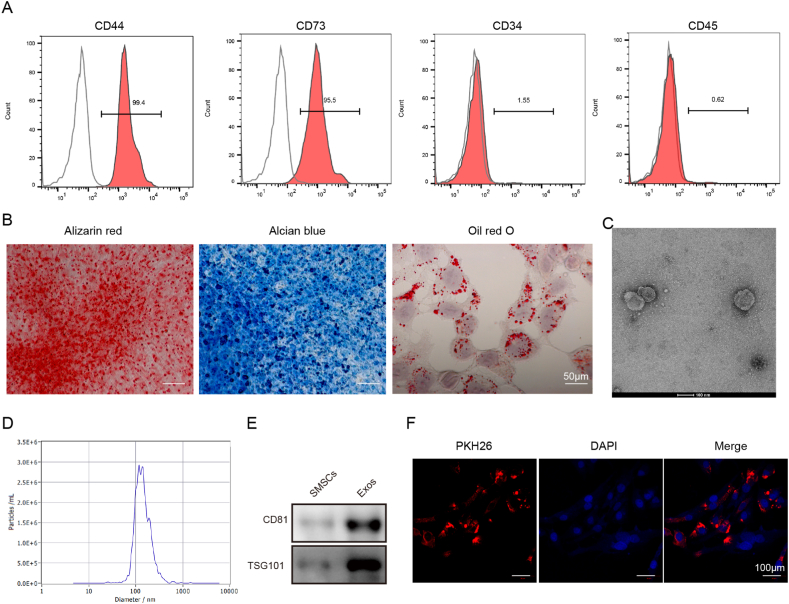
Identification of SMSCs and SMSC-derived Exos. (A) The percentage of CD44^+^, CD73^+^, CD34^−^, and CD45^−^ SMSCs was analyzed by flow cytometry. (B) The multilineage differentiation abilities of SMSCs were evaluated by Alizarin Red S, Oil Red O, and Alcian Blue staining. Scale bar = 50 μm. (C) The morphology of Exos was examined by TEM. (D) Light scattering analysis of the size of the isolated Exos. (E) Western blotting analysis of the marker proteins (CD81 and TSG101) of Exos. (F) Cartilage cells were treated with PKH67-labeled Exos, and exosome uptake by SMSCs was observed by fluorescence microscopy. Scale bar = 100 μm. (For interpretation of the references to colour in this figure legend, the reader is referred to the Web version of this article.)

For the identification of SMSCs, the proportion of CD44^+^, CD73^+^, CD34^−^, and CD45^−^SMSCs was evaluated by flow cytometry. The positive expression of CD44 and CD73 and the negative expression of CD34 and CD45 indicated the presence of SMSCs (Fig. 1A). In addition, the osteogenic and adipogenic differentiation capacities of SMSCs were confirmed by Alizarin Red S and Oil Red O staining (Fig. 1B). Besides, the positive deep blue staining in Alcian blue staining indicated the strong chondrogenic potential of SMSCs (Fig. 1B). Moreover, Exos were isolated from SMSCs, and their single membrane structure was observed by TEM (Fig. 1C). Light scattering analysis showed that the exosome size was approximately 100 nm in diameter (Fig. 1D). In addition, we found the presence of exosome marker proteins (CD81 and TSG101) in Exos but their absence in SMSCs (Fig. 1E, uncropped blots in Fig. S4). Moreover, PKH26-labeled Exos were observed in the cytoplasm of cartilage cells, indicating the internalization of Exos by cartilage cells (Fig. 1F). Therefore, Exos were successfully extracted from SMSCs and could be internalized by cartilage cells.

SMSC-derived Exos restrained proliferative inhibition, apoptosis, extracellular matrix (ECM) degradation, and inflammation in IL-1β-exposed cartilage cells.

**Fig. 2:**
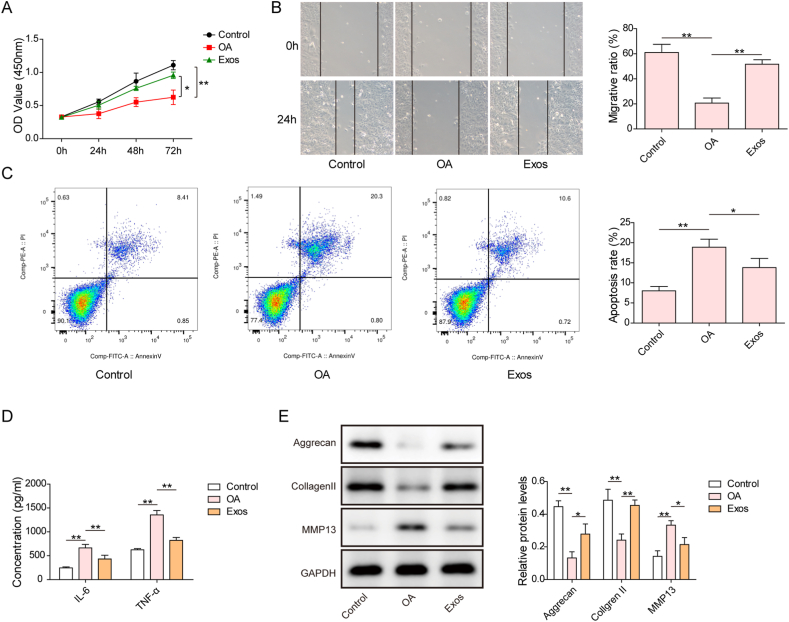
Exos restrained IL-1β-induced cartilage injury *in vitro*. Cartilage cells were pretreated with Exos for 2 h and then treated with IL-1β for 48 h. (A) Cartilage cell proliferation was assessed by CCK-8 assays. (B) Cartilage cell migration was determined by transwell assays. (C) The apoptosis rate of cartilage cells was detected by Annexin V/PI staining on a flow cytometer. (D) IL-6 and TNF-α production was measured by ELISAs. (E) The protein levels of Aggrecan, Collagen II, and MMP13 in cartilage cells were detected by Western blotting. *p < 0.05, **p < 0.01, ***p < 0.001.

We further explored whether Exos could attenuate cartilage damage in an *in vitro* model of OA. The CCK-8 results indicated that IL-1β stimulation remarkably inhibited cartilage cell proliferation, which could be partly restored by Exos (Fig. 2A). Furthermore, the IL-1β-induced decrease in the migratory ability of cartilage cells could be reversed by Exos (Fig. 2B). In addition, apoptosis of cartilage cells triggered by IL-1β was repressed by treatment with Exos (Fig. 2C). IL-1β stimulation enhanced IL-6 and TNF-α release from cartilage cells, whereas Exos effectively restrained the production of IL-6 and TNF-α (Fig. 2D). Additionally, the levels of the ECM secretion-related proteins Aggrecan and Collagen II were decreased, while MMP13 levels were increased in IL-1β-treated cartilage cells, which indicated that IL-1β caused ECM degradation. However, administration of Exos suppressed IL-1β-induced ECM degradation (Fig. 2E, uncropped blots in Fig. S4). These results demonstrated that Exos attenuated cartilage injury in the *in vitro* model of OA.

### 4.2 Treatment with Exos enhanced miR-485-3p expression in cartilage cells

**Fig. 3:**
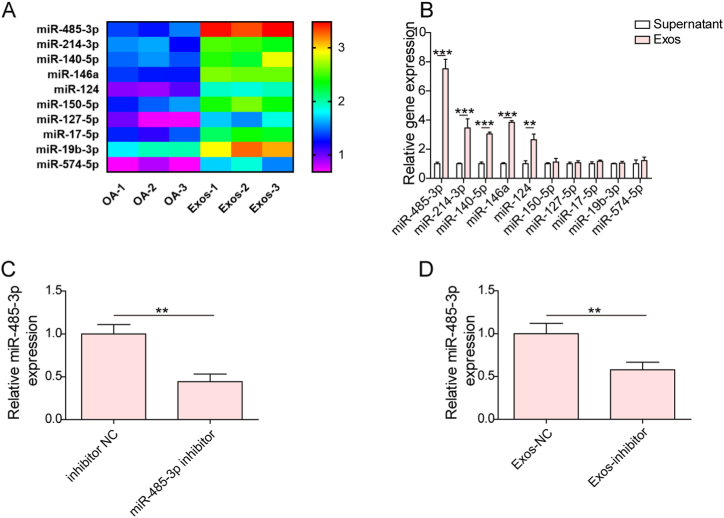
Exos promoted miR-485-3p expression in cartilage cells. (A) The differential expression of miRNAs in cartilage cells after administration of Exos was evaluated by microarray analysis. Ten differentially expressed miRNAs by hierarchical clustering analysis are illustrated. (B) qRT‒PCR analysis of miR-485-3p, miR-214-3p, miR-140-5p, miR-146a, miR-124, miR-150-5p, miR-127-5p, miR-17-5p, miR-19b-3p, and miR-574-5p levels in Exos. (C) & (D) SMSCs were transfected with inhibitor NC or miR-485-3p inhibitor. miR-485-3p expression in SMSCs and their Exos was determined by qRT‒PCR. **p < 0.01, ***p < 0.001.

To explore the protective mechanism through which Exos inhibited OA development, we investigated Exo-induced differentially expressed miRNAs using a miRNA microarray assay. The top 10 differentially expressed miRNAs between the OA and Exo groups are shown in Fig. 3A qRT‒PCR results indicated that miR-485-3p, miR-214-3p, miR-140-5p, miR-146a, and miR-124 levels were higher in Exos than in the supernatant of SMSCs, whereas there were no significant differences in miR-150-5p, miR-127-5p, miR-17-5p, miR-19b-3p, and miR-574-5p levels (Fig. 3B). miR-485-3p exhibited the highest level among the miRNAs; thus, miR-485-3p was the focus of subsequent experiments. In addition, we found that miR-485-3p expression was reduced in both SMSCs and their exosomes after transfection with the miR-485-3p inhibitor (Fig. 3C and D). The above observations revealed that miR-485-3p was transferred by Exos, which might affect cartilage injury *in vitro*.

### 4.3 miR-485-3p silencing weakened the protective effect of Exos on cartilage cells

**Fig. 4:**
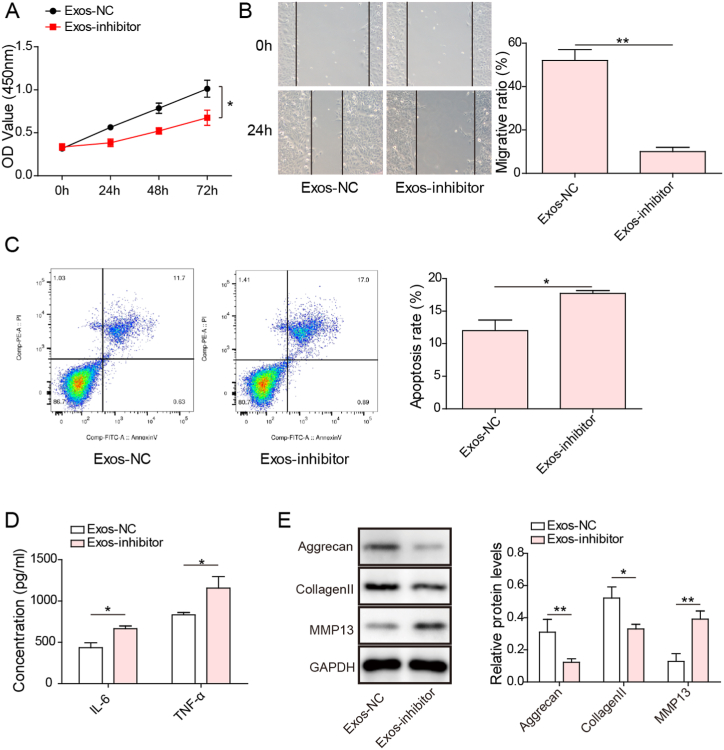
Exos attenuated IL-1β-induced cartilage injury by delivering miR-485-3p. IL-1β-stimulated cartilage cells were treated with Exos derived from inhibitor NC or miR-485-3p inhibitor-transfected SMSCs. (A) Proliferation of cartilage cells was evaluated by CCK-8 assays. (B) Cartilage cell migration was detected by Transwell assays. (C) The percentage of apoptotic cartilage cells was analyzed by flow cytometry. (D) The levels of IL-6 and TNF-α were determined by ELISAs. (E) Western blotting analysis of the protein levels of Aggrecan, Collagen II, and MMP13 in cartilage cells. *p < 0.05, **p < 0.01.

We further verified the involvement of miR-485-3p in Exo-mediated protection against cartilage injury in OA. The proliferation of cartilage cells was restrained in the Exo-inhibitor group (Fig. 4A). The migration of Exo-treated cartilage cells was repressed by the miR-485-3p inhibitor (Fig. 4B). Moreover, apoptosis was triggered in the Exo-inhibitor group (Fig. 4C). Inhibition of miR-485-3p in Exos enhanced IL-6 and TNF-α levels in the supernatant of cartilage cells (Fig. 4D). Accordingly, we found downregulation of Aggrecan and Collagen II protein expression but upregulation of MMP13 expression in the Exo-inhibitor group (Fig. 4E, uncropped blots in Fig. S4). These findings revealed that Exos attenuated cartilage injury by modulating miR-485-3p expression.

### 4.4 NRP1 was a target gene of miR-485-3p

**Fig. 5:**
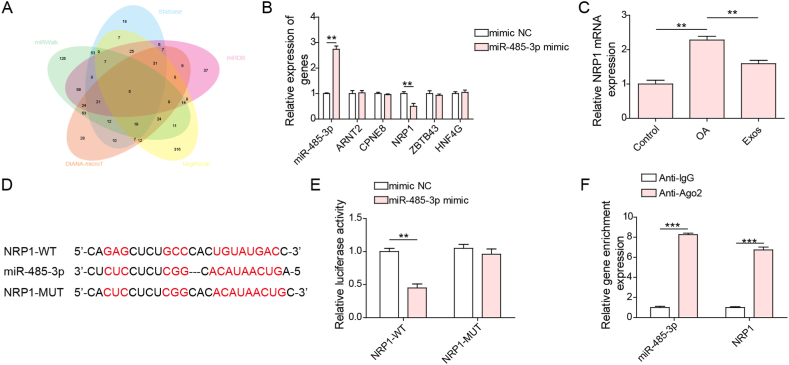
miR-485-3p directly targeted NRP1 in cartilage cells. (A) The potential target genes of mmu-miR-485-3p were predicted by the StarBase, miRDB, miRWalk, DIANA-microT, and TargetScan databases. Five target genes (ARNT2, CPNE8, NRP1, ZBTB43, and HNF4G) were selected by overlapping the prediction results. (B) miR-485-3p, ARNT2, CPNE8, NRP1, ZBTB43, and HNF4G levels were evaluated by qRT‒PCR. (C) qRT‒PCR analysis of NRP1 mRNA expression in cartilage cells. (D) The predicted binding sites of miR-485-3p in the NRP1 3′UTR. The direct interaction between miR-485-3p and NRP1 was confirmed by dual luciferase reporter assays (E) and RIP assays (F). **p < 0.01, ***p < 0.001.

The StarBase, miRDB, miRWalk, DIANA-microT, and TargetScan databases were used to predict the potential target genes of miR-485-3p. By overlapping the prediction results, we identified five target genes (ARNT2, CPNE8, NRP1, ZBTB43, and HNF4G) (Fig. 5A). Furthermore, miR-485-3p overexpression significantly decreased NRP1 expression; however, the expression of the other four target genes was not changed after transfection with miR-485-3p mimics (Fig. 5B). Therefore, NRP1 was selected as the target gene of miR-485-3p. Furthermore, NRP1 levels were elevated in cartilage cells after IL-1β treatment, which was reversed by Exos (Fig. 5C). As shown in Fig. 5D, the binding sites of miR-485-3p on the NRP1 3′UTR were predicted by the starBase database (Fig. 5D). Transfection with miR-485-3p mimics strikingly reduced the luciferase activity of the NRP1-WT group but not that of the NRP1-MUT group (Fig. 5E). In addition, both miR-485-3p and NRP1 were enriched by the Ago-2 antibody (Fig. 5F), suggesting a direct interaction between miR-485-3p and NRP-1. Thus, NRP1 was a target gene of miR-485-3p.

#### 4.4.1 NRP1 overexpression reversed Exo-mediated protection by activating the PI3K/Akt pathway

**Fig. 6:**
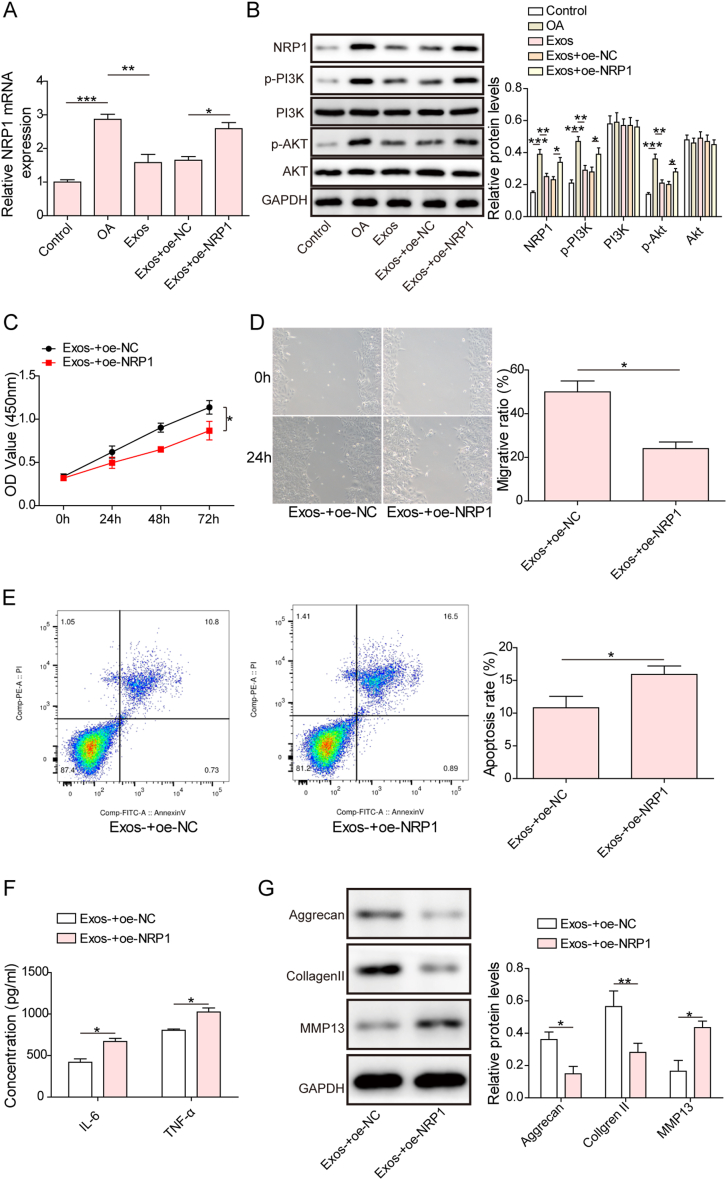
NRP1 activated the PI3K/Akt pathway to reverse Exo-mediated protection. Cartilage cells were transfected with oe-NC or oe-NRP1, followed by treatment with SMSC-derived Exos. (A) The mRNA level of NRP1 was measured by qRT‒PCR. (B) The protein abundance of NRP1, *p*-Akt, Akt, p-PI3K, and PI3K in cartilage cells was evaluated by Western blotting. (C) Cell proliferation was assessed by CCK-8 assays. (D) Cartilage cell migration was determined by Transwell assays. (E) Apoptosis was measured by flow cytometry. (F) The release of IL-6 and TNF-α from cartilage cells was detected by ELISAs. (G) Western blotting analysis of the protein levels of Aggrecan, Collagen II, and MMP13. *p < 0.05, **p < 0.01, ***p < 0.001.

To validate whether NRP1 participated in the beneficial effect of Exos, we transfected cartilage cells with oe-NRP1. The mRNA level of NRP1 was increased by IL-1β treatment, which was reversed by Exos. Transfection with oe-NRP1 significantly increased NRP1 levels in the presence of Exos (Fig. 6A). In addition, NRP1, p-PI3K, and *p*-Akt protein levels were elevated in IL-1β-stimulated cartilage cells, but this change was abrogated by administration of Exos. However, NRP1 overexpression abolished the Exo-mediated changes described above (Fig. 6B, uncropped blots in Fig. S4). Functionally, Exo-induced proliferation, migration, and apoptosis inhibition in cartilage cells could be abolished by NRP1 overexpression (Fig. 6C–E). In addition, the release of IL-6 and TNF-α from cartilage cells in the presence of Exos was enhanced by NRP1 overexpression (Fig. 6F). Enforced expression of NRP1 downregulated Aggrecan and Collagen Ⅱ protein expression and upregulated MMP13 expression in cartilage cells treated with Exos (Fig. 6G, uncropped blots in Fig. S4). In addition, PI3K-IN-1, an inhibitor of the PI3K/AKT pathway, was added to NRP1-overexpressing cartilage cells in the presence of Exos. We found that *p*-Akt and p-PI3K expression was restrained by PI3K-IN-1 (Fig. S1A, uncropped blots in Fig. S5). Moreover, PI3K-IN-1 treatment promoted proliferation and inhibited apoptosis and IL-6 and TNF-α release in cartilage cells (Figs. S1B–D). Aggrecan and Collagen Ⅱ expression was promoted, while MMP13 expression was reduced by PI3K-IN-1 (Fig. S1E, uncropped blots in Fig. S5). As shown in Fig. S2, there were no significant changes in *p*-ERK1/2 and ERK1/2 protein levels among the treatment groups (uncropped blots in Fig. S5). Thus, Exos exerted their protection against osteoarthritic cartilage injury through inactivation of the NRP1/PI3K/Akt pathway.

## 5 Discussion

OA, characterized by degeneration of cartilage, is a frequent degenerative joint disorder worldwide [24]. Unfortunately, due to the unclear and complicated pathogenesis, there is still a lack of specific or effective treatments for OA patients. Therefore, there is an urgent need to develop effective therapies to slow the progression of OA. The novelty of our study lies in that SMSCs-derived Exos transferred miR-485-3p to ameliorate cartilage damage during OA progression. Mechanistically, miR-485-3p targeted NRP1 to inactivate the PI3K/Akt pathway. Our findings provide a theoretical foundation for the development of potential interventions for OA, and SMSCs-derived Exos represent as a solid alternative to existing therapeutics.

Exos derived from osteoarthritic synovial fluid have been confirmed to impede OA development [25]. Exos, as intercellular messengers, are responsible for the repair of osteoarthritic cartilage through paracrine mechanisms [26]. We found that Exos derived from SMSCs clearly promoted proliferation and migration and restrained apoptosis and inflammation in cartilage cells. However, Tao et al. reported the side effects of Exos on decreasing ECM secretion via SOX9 inhibition [11]. Paradoxically, our results showed that ECM protein degradation was restrained by Exos, which might be due to different cell types and cell states.

To uncover the protective mechanism of Exos, we performed a miRNA microarray assay. miR-485-3p with high expression in response to Exos was investigated. Notably, miR-485-3p levels were found to be decreased in the anterior cruciate ligament tissues of OA [27]. Moreover, miR-485-3p overexpression repressed osteoarthritic cartilage cell injury [15]. However, the relationship between highly expressed miR-485-3p in SMSC-Exos and cartilage cell damage remains unclear. In view of this, we hypothesized that miR-485-3p levels are inhibited in SMSC-Exos. Our results showed that inhibition of exosomal miR-485-3p counteracted the beneficial effect of Exos on cartilage cell damage, which proved that Exos conferred protection against osteoarthritic cartilage injury by delivering miR-485-3p.

miRNAs exert their biological functions by suppressing their target gene expression [28]. In this work, NRP1 was confirmed as a target gene of miR-485-3p, and NRP1 expression was restrained by miR-485-3p mimics. Additionally, rescue experiments were performed to validate the role of NRP1 in Exo-mediated protection. We found that enforced expression of NRP1 remarkably abrogated the beneficial effects of SMSC-derived Exos, which implied that NRP1, as a downstream target of exosomal miR-485-3p, participated in the protective mechanism of Exos in OA. Aberrant activation of the PI3K/Akt pathway in articular cartilage leads to cartilage destruction during OA development [29]. Inactivation of the PI3K/Akt pathway has been considered an effective strategy to delay OA progression [30]. Notably, NRP1 has been confirmed to be an activator of the PI3K/Akt pathway [31]. As expected, our results confirmed that Exos caused inactivation of the PI3K/Akt pathway in the *in vitro* OA model, which could be counteracted by NRP1 overexpression. More importantly, treatment with a PI3K/Akt pathway inhibitor weakened NRP1-mediated cartilage damage. These results proved that PI3K/Akt, as a downstream pathway of NRP1, was implicated in the modulatory mechanism of exosomal miR-485-3p in OA.

Cartilage cells were adopted in our study. However, the repetition of experimental results in chondrocytes remains unclear. Therefore, chondrocytes were treated with IL-1β (10 ng/mL) for 48 h to establish an *in vitro* model of OA. We found that treatment with Exos increased miR-485-3p expression but reduced NRP1 expression in IL-1β-exposed chondrocytes (Fig. S3), which was consistent with the results in cartilage cells. In the future, we will validate our findings in chondrocytes to further confirm the conclusion of this work.

## 6 Conclusion

Taken together, the results of this study demonstrated that SMSC-derived exosomal miR-485-3p contributed to proliferation and migration but suppressed apoptosis, inflammation, and ECM degradation of osteoarthritic cartilage cells by targeting NRP1 to inactivate the PI3K/Akt pathway, thereby alleviating OA. This study provides evidence for SMSC-derived Exos as an effective therapy for OA in clinical practice.

## Ethics approval statement

No animal and clinical studies were conducted in this study, and all the cells used in this study were purchased from Procell (Wuhan, China). Therefore, this study does not need the relevant approval of the ethics Committee.

## Consent for publication

Not applicable.

## Data availability statement

Data will be made available on request.

## CRediT authorship contribution statement

**Mingjun Qiu:** Writing – original draft, Formal analysis, Conceptualization. **Yanhua Xie:** Writing – review & editing, Supervision, Methodology. **Guanghua Tan:** Writing – review & editing, Supervision. **Xiaoxu Wang:** Writing – review & editing, Supervision, Investigation. **Peiguan Huang:** Supervision, Conceptualization. **Liang Hong:** Writing – review & editing, Supervision, Conceptualization.

## Declaration of competing interest

The authors declare that they have no known competing financial interests or personal relationships that could have appeared to influence the work reported in this paper.

## Abbreviations

OA: osteoarthritis
Exos: synovial mesenchymal stem cell-derived exosomes
miRNAs: microRNAs
MSCs: mesenchymal stem cells
NRP1: neuropilin-1
SMSCs: mouse synovial-derived mesenchymal stromal cells
FBS: fetal bovine serum
ECM: extracellular matrix
qRT‒PCR: quantitative real-time polymerase chain reaction
CCK-8: Cell Counting Kit-8
PI: propidium iodide
WT: wild type
MUT: mutant
RIP: RNA immunoprecipitation
SD: standard deviation
ANOVA: analysis of variance
