# Supplementary material for: Synovial mesenchymal stem cell-derived exosomal miR-485-3p relieves cartilage damage in osteoarthritis by targeting the NRP1-mediated PI3K/Akt pathway: Exosomal miR-485-3p relieves cartilage damage
Source: Heliyon. 2024 Jan 3;10(2):e24042. doi: 10.1016/j.heliyon.2024.e24042 (PMC10826677; doi:10.1016/j.heliyon.2024.e24042)
Supplement: Multimedia component 1 [file mmc1.docx]

**Supplementary figure legends**


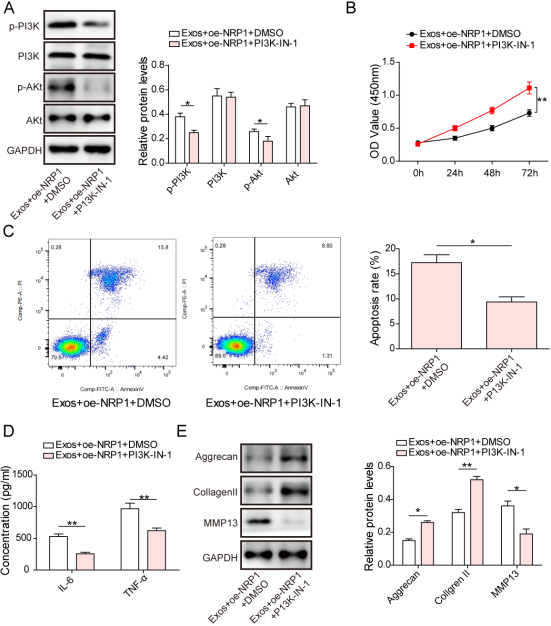


**Figure S1** Inactivation of PI3K/Akt pathway NRP1-mediated cartilage damage. NRP1-overexpressed cartilage cells were treated with PI3K/AKT pathway inhibitor PI3K-IN-1 (25 μM) in the presence of Exos. (A) The protein levels of p-Akt, Akt, p-PI3K, PI3K in cartilage cells was evaluated by Western blotting. (B) Cell proliferation was detected by CCK-8. (C) Apoptosis of cartilage cells was assessed by flow cytometry. (D) IL-6 and TNF-α levels in the supernatant of cartilage cells were detected by ELISA. (E) Western blotting analysis of the protein levels of Aggrecan, Collagen II, and MMP13. *p < 0.05, **p < 0.01, ***p < 0.001.


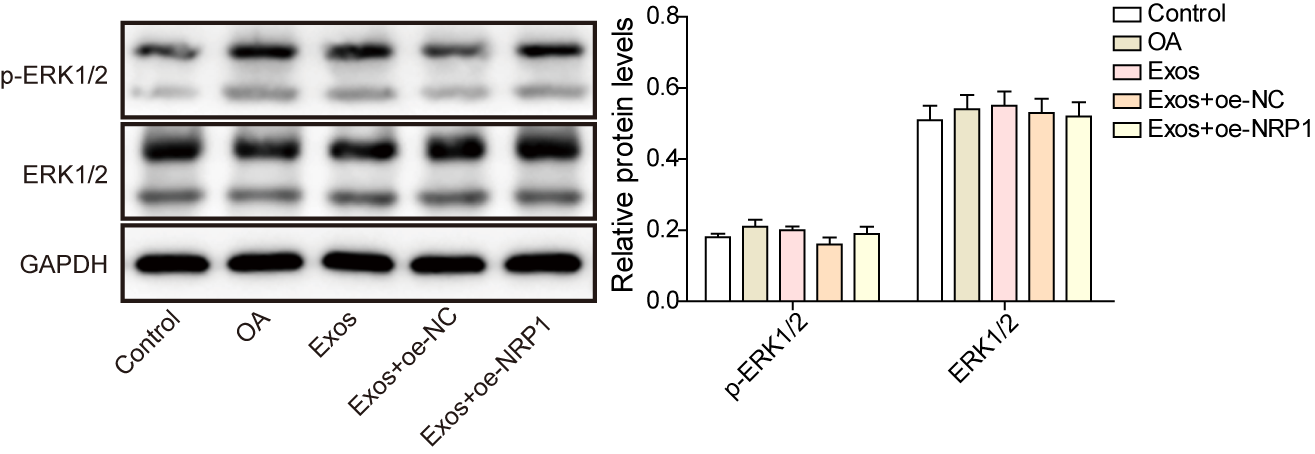


**Figure S2** Exos-treated cartilage cells were transfected with oe-NC or oe-NRP1. The protein levels of p-ERK1/2, ERK1/2 were determined by Western blotting. *p < 0.05, **p < 0.01, ***p < 0.001.


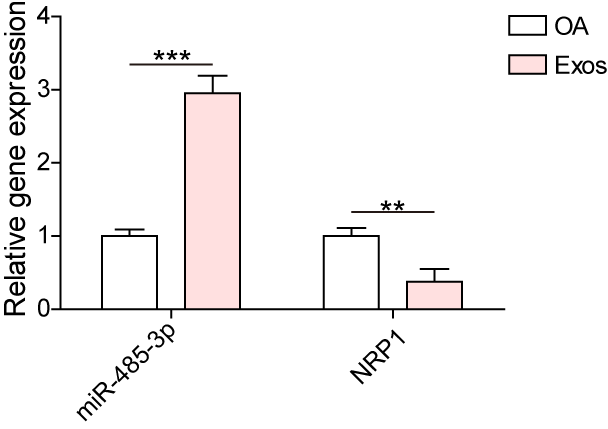


**Figure S3** The mouse primary chondrocytes were pretreated with Exos for 2 h and then stimulated with IL-1β. The levels of miR-485-3p and NRP1 in chondrocytes were detected by qRT-PCR. *p < 0.05, **p < 0.01, ***p < 0.001.


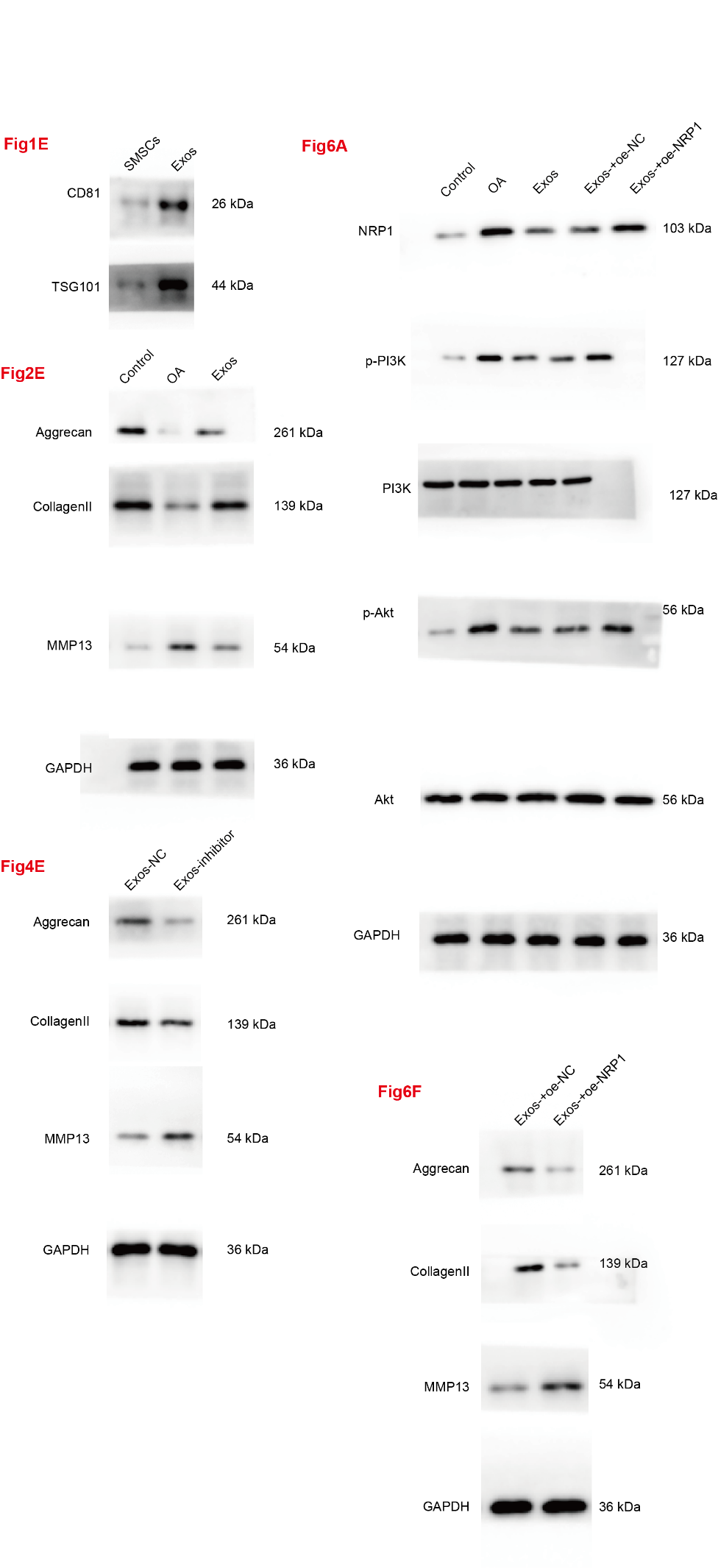


**Figure S4** Uncropped blots of Figure 1-6.


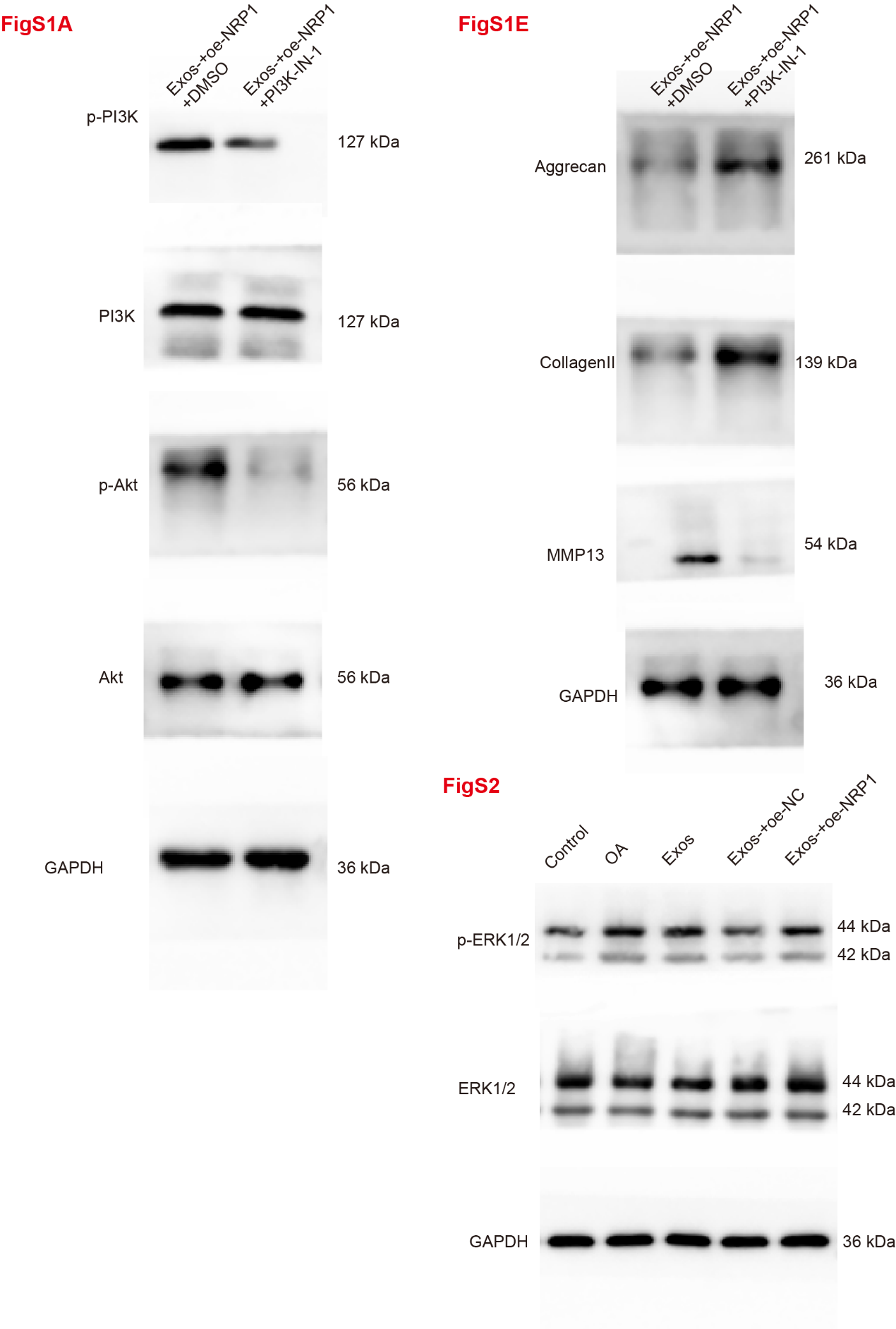


**Figure S5** Uncropped blots of Figure S1-2.
